# Aberdeen Lunatic Hospital

**Published:** 1831

**Authors:** 


### ABERDEEN LUNATIC HOSPITAL.

#### CASE OF INSANITY AND PARALYSIS, WITH DISSECTION.

PETER KEITH, ætatis 66, a carter, formerly in the navy, admitted 2d Sept. 1829.

About eighteen months ago he became gradually affected with insanity. Imagines himself to be Admiral Keith, and related to the Royal family and indulges in other delusions of a similar description. He has been occasionally violent, requiring restraint—was much addicted to the use of spirits and frequently drank to intoxication.

19th July, 1830. His delusions still exist, but without the least excitement. Can answer questions at times coherently, but seems stupid and greatly enfeebled in his intellect; and has a good deal of stammering and difficulty in articulating. For the last two months he has laboured under a partial loss of power of both lower extremities, which is reported to have come on gradually. Pulse very feeble. He cannot rise from bed, nor move the limbs, further than drawing them a little upwards with much exertion. No complaint of pain or uneasiness in any region of the body.

27th Jan. 1831. During his confinement to bed he retained his appetite to within a day or two of his decease, and suffered no pain, unless from occasional tympanitic distention of the abdomen, relieved by laxatives and aromatic tincture. His feet and legs became extensively œdematous at one time, but this also disappeared and did not recur. About a month or two before his death he experienced the same partial loss of muscular power of the superior extremities. His pulse was generally feeble and bowels torpid. He was allowed a nourishing diet and tumbler of porter daily.

The symptoms having varied little from the date of the above report, he died this day.

*Dissection* 36 *hours after death.* The body was full and muscular and well covered with fat; his appetite having continued good to the last.

The calvarium being firmly adherent to the dura mater produced in its removal a laceration of the latter and allowed a considerable quantity of serous fluid to escape. There was a copious gelatinous deposit over the entire surface of the hemispheres between the pia mater and arachnoid, and forming a layer of considerable thickness more especially over the anterior lobes intermixed with some serous fluid.

The latter membrane was quite opaque and probably thickened. The substance of the brain as well as the membranes were by no means vascular.

There was situated between the middle and posterior lobes and immediately under the tunics a small irregular-shaped cavity with vascular parietes, without distinct boundaries, containing the debris of softened and broken down cortical substance. The ramollissement extended into the medullary substance where it gradually disappeared in the surrounding healthy structure.

There was a most decided tendency to a general ramollissement of the cineritious matter generally, and of the central parts of the organ more especially.

The medullary substance, was comparatively free from this disorganising process.

The ventricles were greatly distended with fluid. The lungs were quite sound, with the exception of a small mass of crude tubercles situated in the apex of the right superior lobe.

The parietes of the left ventricle of the heart were considerably hypertrophied, and those of the right were, on the other hand, much attenuated; in some parts entirely deprived of muscular fibre, and reduced to a thin layer of adipose tissue. The liver was rather friable. Other viscera healthy.

The foregoing appears to be a pretty well marked case of what Georget terms "chronic muscular paralysis," and corresponding in its pathology (as proved by dissection) with the general description of this class of cases as given by the above author and noticed by Dr. Burrows in his ninth Commentary on Insanity.

The exciting causes were such as tended to induce repeated vascular excitement and such as would ultimately tend no doubt to terminate in a permanent chronic inflammatory action in some region or organ of the body.

The ramollissement may therefore, in this case at least, be fairly considered as the effects of vascular action.—J. M.
*Lunatic Asylum, Aberdeen,*
31st Jan. 1831.

N.B. It is to be hoped that the medical officers of lunatic hospitals will take advantage of their situations and furnish their professional brethren with records of the phenomena and pathology of insanity — records which are much wanted in this country.—Ed.

---

XXVI.

